# A Multivariable Prediction Model for Mild Cognitive Impairment and Dementia: Algorithm Development and Validation

**DOI:** 10.2196/59396

**Published:** 2024-11-22

**Authors:** Sarah Soyeon Oh, Bada Kang, Dahye Hong, Jennifer Ivy Kim, Hyewon Jeong, Jinyeop Song, Minkyu Jeon

**Affiliations:** 1 Institute of Global Engagement & Empowerment Yonsei University Seoul Republic of Korea; 2 Mo-Im Kim Nursing Research Institute Yonsei University College of Nursing Seoul Republic of Korea; 3 Institute for Innovation in Digital Healthcare Yonsei Univeristy Seoul Republic of Korea; 4 College of Nursing and Brain Korea 21 FOUR Project Yonsei University Seoul Republic of Korea; 5 Department of Electrical Engineering and Computer Science Massachusetts Institute of Technology Cambridge, MA United States; 6 Department of Physics Massachusetts Institute of Technology Cambridge, MA United States; 7 Department of Computer Science Princeton University New Jersey, NJ United States

**Keywords:** mild cognitive impairment, machine learning algorithms, sociodemographic factors, gerontology, geriatrics, older people, aging, MCI, dementia, Alzheimer, cognitive, machine learning, prediction, algorithm

## Abstract

**Background:**

Mild cognitive impairment (MCI) poses significant challenges in early diagnosis and timely intervention. Underdiagnosis, coupled with the economic and social burden of dementia, necessitates more precise detection methods. Machine learning (ML) algorithms show promise in managing complex data for MCI and dementia prediction.

**Objective:**

This study assessed the predictive accuracy of ML models in identifying the onset of MCI and dementia using the Korean Longitudinal Study of Aging (KLoSA) dataset.

**Methods:**

This study used data from the KLoSA, a comprehensive biennial survey that tracks the demographic, health, and socioeconomic aspects of middle-aged and older Korean adults from 2018 to 2020. Among the 6171 initial households, 4975 eligible older adult participants aged 60 years or older were selected after excluding individuals based on age and missing data. The identification of MCI and dementia relied on self-reported diagnoses, with sociodemographic and health-related variables serving as key covariates. The dataset was categorized into training and test sets to predict MCI and dementia by using multiple models, including logistic regression, light gradient-boosting machine, XGBoost (extreme gradient boosting), CatBoost, random forest, gradient boosting, AdaBoost, support vector classifier, and k-nearest neighbors, and the training and test sets were used to evaluate predictive performance. The performance was assessed using the area under the receiver operating characteristic curve (AUC). Class imbalances were addressed via weights. Shapley additive explanation values were used to determine the contribution of each feature to the prediction rate.

**Results:**

Among the 4975 participants, the best model for predicting MCI onset was random forest, with a median AUC of 0.6729 (IQR 0.3883-0.8152), followed by k-nearest neighbors with a median AUC of 0.5576 (IQR 0.4555-0.6761) and support vector classifier with a median AUC of 0.5067 (IQR 0.3755-0.6389). For dementia onset prediction, the best model was XGBoost, achieving a median AUC of 0.8185 (IQR 0.8085-0.8285), closely followed by light gradient-boosting machine with a median AUC of 0.8069 (IQR 0.7969-0.8169) and AdaBoost with a median AUC of 0.8007 (IQR 0.7907-0.8107). The Shapley values highlighted pain in everyday life, being widowed, living alone, exercising, and living with a partner as the strongest predictors of MCI. For dementia, the most predictive features were other contributing factors, education at the high school level, education at the middle school level, exercising, and monthly social engagement.

**Conclusions:**

ML algorithms, especially XGBoost, exhibited the potential for predicting MCI onset using KLoSA data. However, no model has demonstrated robust accuracy in predicting MCI and dementia. Sociodemographic and health-related factors are crucial for initiating cognitive conditions, emphasizing the need for multifaceted predictive models for early identification and intervention. These findings underscore the potential and limitations of ML in predicting cognitive impairment in community-dwelling older adults.

## Introduction

Mild cognitive impairment (MCI) is the transitional phase between normal aging and dementia [1]. According to a systematic review of global prevalence, MCI is estimated to affect approximately 15.56% of community-dwelling adults aged 50 years and older [2]. The annual progression rate of MCI to dementia, which is characterized by acquired memory loss that interferes with daily functioning [3], is believed to range from 12% to 17% [1]. Given that dementia progression incurs substantial social, financial, and health care costs for patients and caregivers, its economic impact, encompassing unpaid caregiving and nursing home placement, is estimated to reach US $800 billion worldwide [1].

Currently, there is no cure for dementia; however, early detection and intervention for MCI can mitigate the risk of dementia. Early detection of MCI is important for optimizing patient care, potentially enabling the timely use of disease-modifying therapies to prevent Alzheimer disease (AD) and related dementia [4]. As the life expectancy of patients with AD is approximately 8-10 years after symptom onset, preventing disease progression at an early stage is crucial [3]. Early identification of MCI facilitates the treatment of reversible underlying conditions and the prevention of the behavioral and psychological symptoms of dementia. This provides an opportunity to control lifestyle-related diseases, thereby potentially preventing or slowing dementia progression [5]. Additionally, it offers family members and caregivers critical preparation time for advanced care planning and social support, potentially reducing overall treatment costs and caregiver distress [6].

However, the early detection of MCI faces challenges, including underdiagnosis or misdiagnosis in clinical settings, limited physician training and support, challenges within health care systems, and difficulties associated with diagnostic test design and validation [5]. Since MCI symptoms in the early stages can vary significantly among individuals, cognitive tests should account for the diverse etiologies and varying demands of day-to-day living within patient populations [7]. Primary care visits, typically lasting 10 minutes or less, may have limitations in conducting accurate and thorough diagnostic assessments, and few measures have been validated for various races, ethnicities, and languages [7]. Adding to these challenges is the ethical dilemma for physicians in disclosing a dementia diagnosis, particularly among sociodemographic groups where factors such as cultural beliefs or socioeconomic status may contribute to diagnostic uncertainty and increase the risk of ambiguity and potential misdiagnosis [8].

The high prevalence of memory disturbances in older adults makes it difficult to diagnose MCI accurately. Many older adults frequently report incidents of “subjective memory impairment” (SMI); however, recent studies have confirmed that among such individuals, many may have other reasons for SMI, such as depression, despite being cognitively healthy [9]. For example, in a previous study of 5511 adults aged 70 years and older, 50% experienced SMI, and only 17% screened positive for dementia [9]. These findings indicate that the SMI may be an inadequate criterion for determining whether an individual should be tested for MCI or dementia in an extensive and time-consuming manner. Similarly, only 10% to 20% of older community populations with subjective cognitive impairment report memory loss, making it difficult to establish an accurate and consistent gold standard for MCI detection, which further complicates the diagnostic process in clinical settings [9].

Nevertheless, the increasing use of predictive models with machine learning (ML) algorithms that can process complex data, from sociodemographic and health-related factors to extensive neuropsychological tests and biomarkers, has encouraged researchers to investigate the potential of ML in identifying individuals with MCI and dementia [10]. In dementia risk prediction, traditional statistical methods, such as Cox and logistic regression analyses, are typically used under the assumption of linear relationships between variables; however, they lack sufficient external or internal validation, indicating the need to use larger and more diverse datasets for better accuracy [11]. Against this backdrop, newer algorithms that incorporate ML algorithms are emerging as promising alternatives, potentially offering comparable levels of accuracy, particularly when a wide range of data is available [12].

Scholars are increasingly exploring neuropsychological assessments and diverse biomarkers, including imaging data (magnetic resonance imaging and positron emission tomography), laboratory tests (blood and cerebrospinal tests), and genetic data to assess whether ML systems can revolutionize MCI and AD treatments [12]. In frequently used datasets, such as the Australian Imaging, Biomarker, and Lifestyle [12] and Open Access Series of Imaging Studies [13] from Washington University’s Alzheimer’s Disease Research Center, multiple studies have emerged that incorporate common tests, such as the Mini-Mental State Exam, neuropsychiatric inventory, and health-related quality of life, along with biomarker and neuroimaging data in ML models to enhance classification accuracy [12]. Despite these efforts, ML expert systems have not yet been incorporated into everyday practice, and the classification of patients with MCI and dementia remains unreliable [12]. Limited research exists on how ML algorithms can be used in real-life settings for clinician decision-making and the support of community-dwelling older adults [12]. To address this gap, identifying individuals at risk of developing MCI and AD based on their sociodemographic and health-related characteristics is essential. This approach, which uses readily available information, offers more cost-effective alternatives than biomarker measurements for targeting at-risk individuals [14]. Furthermore, additional studies using ML with sociodemographic features are necessary to enhance the applicability of these models across various populations [15].

The Republic of Korea presents a unique context for this study, with one of the fastest aging populations and the lowest birth rates globally; approximately 20% of its population is expected to be aged 65 years or older by 2025 [16]. Epidemiologically, more than 3 million older adults in South Korea are predicted to develop dementia in 2050, which is a 16% increase from current statistics that show that as of 2021, approximately 10.4% (8.6 million individuals) and 22.7% of older adults aged 65 years and older will develop dementia and MCI, respectively [17]. Currently, national costs of dementia care make up about 0.9% of the national gross domestic product (KRW 18.7 trillion [equivalent to US $14.4 billion]) and have been rising annually by around 5% [17]. Considering that the global prevalence of dementia was 57.4 million in 2019 and is estimated to increase to 152.8 million by 2050, this will mean that South Korea is responsible for approximately 2% of global dementia cases due to population aging [18].

Due to such factors, as well as a significant increase in AD-related mortality, national initiatives such as the “national responsibility for dementia care” aim to structure a more efficient dementia care infrastructure, reduce the care burden among individuals living with dementia and their families, and design preventative measures for early identification to mitigate dementia risk [19]. One of the country’s largest population-based prospective cohort studies of middle-aged and older adults, the Korean Longitudinal Study of Aging (KLoSA), was established in 2005 by the Korean Employment Insurance Fund of the Ministry of Labor to address trends related to rapid population aging [20]. The KLoSA provides detailed information on the demographics, family relationships, health utilization behaviors, and financial status of over 10,000 middle-aged and older adults in the Republic of Korea [21], and its data are increasingly being used to investigate how ML approaches can improve the well-being of these demographics [22]. However, the application of ML in predicting MCI [20] and dementia [23] using the KLoSA data remains limited and varies in terms of methodology [24].

This study used KLoSA data, focusing on lifestyle factors, sociodemographic variables, and common tests, to compare the predictive accuracy of ML-based approaches. Thus, we hope to develop a cost-effective and efficient ML model for MCI and dementia prediction, addressing the crucial gap in real-world applicability to community-dwelling aging populations.

## Methods

### Data Source and Sample

This study used KLoSA data from 2018 to 2020. The KLoSA, established in 2000, is a biennial panel survey that addresses the economic and social challenges stemming from Korea’s transition to an aging society. The core aim of this study was to investigate the evolving aspects of demographics, familial connections, health conditions, health care practices, employment status, and financial dynamics among middle-aged and older Korean adults. A total of 6171 baseline households were surveyed for the KLoSA using multistage stratified sampling based on geographic region (as per the Republic of Korea National Census) and household type (apartment or house). The survey relied on computer-assisted personal interviewing techniques to track the characteristics of baseline participants over time, enabling automatic cognition-related question calculation and scoring [25]. Detailed information on this study is available on the KLoSA website [26].

Participants aged 60 years or older were included in the analyses for this investigation. Individuals without missing information on sociodemographic variables, including sex, homeownership, or self-reported diagnoses of MCI or dementia, were included in the analysis (n=4975). The TRIPOD+AI (Transparent Reporting of a Multivariable Prediction Model for Individual Prognosis or Diagnosis+Artificial Intelligence) checklist was used to guide our study and is available in Multimedia Appendix 1.

### Study Variables

MCI and dementia were identified based on the responses to the question, “Were you diagnosed with dementia?” Responses included (1) “Yes,” (2) “Mild Cognitive Impairment,” and (3) “No.” Individuals answering (1) “Yes” were categorized as having dementia, whereas those selecting (2) “Mild Cognitive Impairment” were categorized as having MCI. Our study focused on predicting the future onset of MCI and dementia among individuals who did not report these conditions in 2018, with predictions extending to 2020. Multiple sociodemographic variables, including sex, age, educational attainment, living arrangements, marital status, and region of residence, were included as covariates in the multivariate analyses for both the traditional and ML models.

### Association Analyses

Outcomes of interest were MCI and dementia diagnoses, with adjusted covariates including sex, age, educational attainment, living arrangements, marital status, region of residence, health-related variables (eg, drinking status, smoking status, and handgrip strength), and functional status (eg, activities of daily living [ADL] and instrumental activities of daily living [IADL] scores). For each model used in the analyses, we ensured robust adjustment for these covariates. Categorical variables underwent rigorous one-hot encoding to facilitate compatibility with our ML models, transforming them into binary columns that effectively captured their multidimensional nature in predictive analytics.

### Feature Selection and Encoding

The features included in our predictive models were selected based on their relevance to dementia and MCI prediction, informed by existing literature and expert opinion [27]. The selected features encompass demographic, socioeconomic, health, and lifestyle variables known to be associated with dementia risk. Specifically, the features used include family, age, education, assets, long-term care (LTC), LTC service, marital status, social engagement, region, ADL, IADL, obesity, drinking, smoking, handgrip strength, comorbidities, falls, pain, and exercise. To prepare these features for the model, categorical variables were encoded using one-hot encoding, significantly increasing the total number of features in the dataset.

### Statistical Analyses

**Figure 1:**
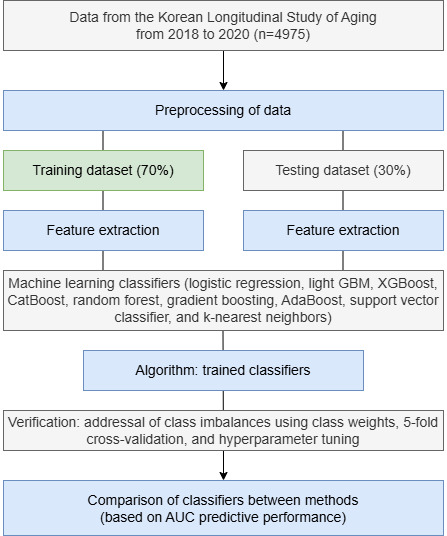
Flow diagram for the investigation. AUC: area under the receiver operating characteristic curve; GBM: gradient-boosting machine; XGBoost: extreme gradient boosting.

Descriptive statistics were used to summarize the baseline sample characteristics (Tables 1 and 2) and frequencies of MCI and dementia (n and %). To address longitudinal and sampling biases stemming from systemic sampling, the KLoSA provided weights for each wave, ensuring unbiased parameter estimates. For the computational analysis, 70% (3483/4975) of the cases were randomly chosen for training, whereas the remaining 30% (1492/4975) were used as test datasets to predict MCI and dementia. By splitting the data into training and testing sets, the models were trained on one subset and evaluated for performance accuracy (Figure 1). Multiple models, including logistic regression, light gradient-boosting machine (GBM), XGBoost (extreme gradient boosting), CatBoost, random forest, gradient boosting, AdaBoost, support vector classifier, and k-nearest neighbors, were used to evaluate predictive performance. Hyperparameters such as the learning rate, maximum depth, and number of estimators were specified for all boosting models to optimize generalization, improve model performance, and produce robust and stable models [28].

**Table 1:** Baseline characteristics of participants according to MCI^a^ onset (2018).

		MCI onset			*P* value
		Yes (n=23)	No (n=4952)		
**Age (years; n=4975), mean (SD)**		80.74 (9.02)	71.13 (8.17)	<.001	
**Sex, n (%)**					.11
	Male (n=2863)	17 (0.59)	2846 (99.41)		
	Female (n=2112)	6 (0.28)	2106 (99.72)		
**Marital status, n (%)**					<.001
	Married (n=3646)	10 (0.27)	3636 (99.73)		
	Widowed (n=1180)	13 (1.10)	1167 (98.90)		
	Single (n=149)	0 (0)	149 (100)		
**Educational attainment, n (%)**					.06
	≤Elementary school (n=2260)	17 (0.75)	2243 (99.25)		
	Middle school (n=883)	2 (0.23)	881 (99.77)		
	High school (n=1366)	3 (0.22)	1363 (99.78)		
	≥University (n=466)	1 (0.21)	465 (99.79)		
**Living arrangements, n (%)**					.58
	Living alone (n=874)	3 (0.34)	871 (99.66)		
	Living with partner (n=2821)	12 (0.43)	2809 (99.57)		
	Living with two or more people (n=1280)	8 (0.63)	1272 (99.38)		
**Region, n (%)**					.42
	Rural (n=3620)	15 (0.41)	3605 (99.59)		
	Urban (n=1355)	8 (0.59)	1347 (99.41)		
**Assets (n=4975), mean (SD)**		2.87 (0.46)	2.34 (0.81)	.006	
**LTC^b^ insurance, n (%)**					.01
	Unaware (n=1961)	16 (0.82)	1945 (99.18)		
	Aware but not in use (n=2948)	7 (0.24)	2941 (99.76)		
	In use (n=66)	0 (0)	66 (100)		
**LTC home service, n (%)**					.62
	Use (n=4923)	23 (0.47)	4900 (99.53)		
	Do not use (n=52)	0 (0)	52 (100)		
**Social engagement frequency, n (%)**					.47
	Daily or weekly (n=2113)	9 (0.43)	2104 (99.57)		
	Monthly (n=1851)	7 (0.38)	1844 (99.62)		
	Yearly or less (n=1011)	7 (0.69)	1004 (99.31)		
**ADL^c^ (n=4975), mean (SD)**		0.87 (2.20)	0.16 (0.94)	<.001	
**IADL^d^ (n=4975), mean (SD)**		3.26 (4.04)	0.53 (1.85)	<.001	
**Handgrip strength, n (%)**					.91
	Increased or stayed the same (n=2437)	11 (0.45)	2426 (99.55)		
	Decreased (n=2538)	12 (0.47)	2526 (99.53)		
**Drinking status, n (%)**					.08
	Yes (n=1466)	3 (0.20)	1463 (99.80)		
	No (n=3509)	20 (0.57)	3489 (99.43)		
**Smoking status, n (%)**					.47
	Yes (n=426)	1 (0.23)	425 (99.77)		
	No (n=4549)	22 (0.48)	4527 (99.52)		
**Frequent exercise, n (%)**					.22
	Yes (n=1685)	5 (0.30)	1680 (99.70)		
	No (n=3290)	18 (0.55)	3272 (99.45)		
**Obesity, n (%)**					.44
	Obese or overweight (n=2742)	12 (0.44)	2730 (99.56)		
	Normal (n=2051)	9 (0.44)	2042 (99.56)		
	Underweight (n=182)	2 (1.10)	180 (98.90)		
**Comorbidity, n (%)**					.72
	0 (n=4948)	23 (0.46)	4925 (99.54)		
	1 or more (n=27)	0 (0)	27 (100)		
**Experience of fall, n (%)**					.02
	Yes (n=93)	2 (2.15)	91 (97.85)		
	No (n=4882)	21 (0.43)	4861 (99.57)		
**Pain in everyday life, n (%)**					<.001
	Yes (n=1368)	15 (1.10)	1353 (98.90)		
	No (n=3607)	8 (0.22)	3599 (99.78)		
**Total (n=4975), n (%)**		23 (0.46)	4952 (99.54)		

^a^MCI: mild cognitive impairment.

^b^LTC: long-term care.

^c^ADL: activities of daily living.

^d^IADL: instrumental activities of daily living.

**Table 2:** Baseline characteristics of participants according to dementia onset (2018).

			Dementia onset				*P* value	
			Yes (n=70)		No (n=4905)			
**Age (years; n=4975), mean (SD)**			81.99 (6.61)		72.03 (8.13)	<.001		
**Sex, n (%)**								.06
	Male (n=2863)	48 (1.68)		2815 (98.32)				
	Female (n=2112)	22 (1.04)		2090 (98.96)				
**Marital status, n (%)**								<.001
	Married (n=3646)	39 (1.07)		3607 (98.93)				
	Widowed (n=1180)	30 (2.54)		1150 (97.46)				
	Single (n=149)	1 (0.67)		148 (99.33)				
**Educational attainment, n (%)**								<.001
	≤Elementary school (n=2260)	57 (2.52)		2203 (97.48)				
	Middle school (n=883)	7 (0.79)		876 (99.21)				
	High school (n=1366)	6 (0.44)		1360 (99.56)				
	≥University (n=466)	0 (0)		466 (100)				
**Living arrangements, n (%)**								.14
	Living alone (n=874)	18 (2.06)		856 (97.94)				
	Living with partner (n=2821)	33 (1.17)		2788 (98.83)				
	Living with two or more people (n=1280)	19 (1.48)		1261 (98.52)				
**Region, n (%)**								.03
	Rural (n=3620)	43 (1.19)		3577 (98.81)				
	Urban (n=1355)	27 (1.99)		1328 (98.01)				
**Assets (n=4975), mean (SD)**			2.67 (0.63)		2.33 (0.81)	.58		
**LTC^a^ insurance, n (%)**								<.001
	Unaware (n=1961)	43 (2.19)		1918 (97.81)				
	Aware but not in use (n=2948)	25 (0.85)		2923 (99.15)				
	In use (n=66)	2 (3.03)		64 (96.97)				
**LTC home service, n (%)**								.13
	Use (n=4923)	68 (1.38)		4855 (98.62)				
	Do not use (n=52)	2 (3.85)		50 (96.15)				
**Social engagement frequency, n (%)**								<.001
	Daily or weekly (n=2113)	28 (1.33)		2085 (98.67)				
	Monthly (n=1851)	12 (0.65)		1839 (99.35)				
	Yearly or less (n=1011)	30 (2.97)		981 (97.03)				
**ADL^b^ (n=4975), mean (SD)**			1.44 (2.53)		0.15 (0.89)	<.001		
**IADL^c^ (n=4975), mean (SD)**			3.19 (4.24)		0.51 (1.79)	<.001		
**Handgrip strength, n (%)**								.30
	Increased or stayed the same (n=2437)	30 (1.23)		2407 (98.77)				
	Decreased (n=2538)	40 (1.58)		2498 (98.42)				
**Drinking status**, **n (%)**								<.001
	Yes (n=1466)	7 (0.48)		1459 (99.52)				
	No (n=3509)	63 (1.80)		3446 (98.20)				
**Smoking status, n (%)**								.39
	Yes (n=426)	4 (0.94)		422 (99.06)				
	No (n=4549)	66 (1.45)		4483 (98.55)				
**Frequent exercise, n (%)**								
	Yes (n=1685)	8 (0.47)		1677 (99.53)		<.001		
	No (n=3290)	62 (1.88)		3228 (98.12)				
**Obesity, n (%)**								.002
	Obese or overweight (n=2742)	34 (1.24)		2708 (98.76)				
	Normal (n=2051)	28 (1.37)		2023 (98.63)				
	Underweight (n=182)	8 (4.40)		174 (95.60)				
**Comorbidity, n (%)**								.53
	0 (n=4948)	70 (1.41)		4878 (98.59)				
	1 or more (n=27)	0 (0)		27 (100)				
**Experience of fall, n (%)**								.78
	Yes (n=93)	1 (1.08)		92 (98.92)				
	No (n=4882)	69 (1.41)		4813 (98.59)				
**Pain in everyday life, n (%)**								<.001
	Yes (n=1368)	34 (2.49)		1334 (97.51)				
	No (n=3607)	36 (1.00)		3571 (99)				
**Total (n=4975), n (%)**			70 (1.41)		4905 (98.59)			

^a^LTC: long-term care.

^b^ADL: activities of daily living.

^c^IADL: instrumental activities of daily living.

### Class Imbalance Methods

Our dataset exhibited significant class imbalance in the target variables “MCI” and “DEMENTIA.” Specifically, the “MCI” variable consisted of 4952 instances of no MCI (Class 0) and only 23 instances of MCI (Class 1), resulting in a highly imbalanced ratio of approximately 215:1. Similarly, the “DEMENTIA” variable included 4905 instances of no dementia (Class 0) and 70 instances of dementia (Class 1), with a class imbalance ratio of approximately 70:1. To address this issue, we analyzed our results using class weights in our ML models using the *class_weight* parameter available in *scikit-learn*, to assign a higher weight to the minority class and a lower weight to the majority class, to penalize the model more for misclassifying minority class instances, and encourage the model to mitigate the impact of class imbalance during training.

### Model Evaluation Procedure

To evaluate the predictive performance of the models, we used a stratified k-fold cross-validation approach with bootstrapping. The data were divided into 5 stratified folds to ensure that each fold contained a representative proportion of the target classes. For each iteration, we trained the models on four of the folds and validated them on the remaining fold. This process was repeated five times, allowing each fold to serve as a test set once. In addition to cross-validation, we used bootstrapping to derive uncertainty estimates for the performance metrics. Specifically, we resampled the training data with replacement to create multiple datasets, fitting the models to these datasets and evaluating their performance. This approach allowed us to calculate confidence intervals for metrics such as accuracy, precision, recall, and area under the receiver operating characteristic curve (AUC). The receiver operating characteristic curves were generated for each model based on the aggregated predictions across all folds, providing a comprehensive view of model performance. This method is consistent with standard practices in predictive modeling, where receiver operating characteristic curves can be informative even in cross-validated contexts. Hyperparameters for each model were set either to default values or based on prior literature. For models such as XGBoost, light GBM, and CatBoost, we specified key hyperparameters like learning rate, maximum depth, and the number of estimators, which were informed by prior research and empirical evaluation. However, we did not perform a systematic grid search or other automated hyperparameter tuning methods in this analysis.

### Ethical Considerations

All procedures involving human participants were performed in accordance with the ethical standards of the Institutional and National Research Committee and the Declaration of Helsinki (1964) and its later amendments or comparable ethical standards. This study was approved by the Institutional Ethics Board of Yonsei University Hospital (approval 4-2023-1506), and informed consent was not required.

## Results

Tables 1 and 2 present the characteristics of the study population according to MCI and dementia onset. Among the 4975 older adults, 23 older adults had MCI, and 70 older adults had dementia. The MCI population had an average age of 80.74 (SD 9.02) years, and the dementia population had an average age of 81.99 (SD 6.61) years. In comparison, those without MCI had an average age of 71.13 (SD 8.17) years, and those without dementia had an average age of 72.03 (SD 8.13) years.

Table 3 shows the performance evaluation of the ML algorithms for MCI and dementia onset predictions. For the MCI onset model, the best model was random forest (median AUC 0.6729, IQR 0.3883-0.8152; 95% CI 0.505-0.7896), followed by k-nearest neighbors (median AUC 0.5576, IQR 0.4555-0.6761; 95% CI 0.4555-0.6761), and support vector classifier (median AUC 0.5067, IQR 0.3755-0.6389; 95% CI 0.3755-0.6389). For the dementia onset model, the best model was XGBoost (median AUC 0.8185, IQR 0.8085-0.8285; 95% CI 0.8085-0.8285), followed by light GBM (median AUC 0.8069, IQR 0.7969-0.8169; 95% CI 0.7969-0.8169) and AdaBoost (median AUC 0.8007, IQR 0.7907-0.8107; 95% CI 0.7907-0.8107).

For MCI, the most important features based on Shapley additive explanation values were pain in everyday life (mean absolute Shapley additive explanation value: 0.71), being widowed (0.49), living alone (0.47), exercising (0.41), and living with a partner (0.29). Other contributing features included monthly social engagement (0.24), decreased handgrip strength (0.21), being obese or overweight (0.16), and living in an urban region (0.16). For dementia, the most predictive feature was grouped as “other 68 features” (1.02), followed by education at the high school level (0.51), education at the middle school level (0.39), exercising (0.30), and monthly social engagement (0.25). Additional predictors included yearly or less frequent social engagement (0.11), drinking (0.14), LTC insurance not in use (0.11), and IADL (0.09).

**Table 3:** AUC^a^ values and CIs for different models according to MCI^b^ and dementia prediction.

Model	Total population, n	MCI, AUC (95% CI)	Dementia, AUC (95% CI)
Light GBM^c^	4975	0.5338 (0.4566-0.6638)	0.8069 (0.7969-0.8169)
XGBoost^d^	4975	0.4940 (0.3044-0.6125)	0.8185 (0.8085-0.8285)
CatBoost	4975	0.4414 (0.3570-0.5599)	0.7474 (0.7474-0.7640)
Random forest	4975	0.6729 (0.5050-0.7896)	0.7468 (0.7286-0.7568)
Gradient boosting	4975	0.4529 (0.3871-0.5607)	0.7899 (0.7399-0.7939)
AdaBoost	4975	0.5148 (0.3600-0.6380)	0.8007 (0.7907-0.8107)
Support vector classifier	4975	0.5067 (0.3755-0.6389)	0.7414 (0.7316-0.7518)

^a^AUC: area under the receiver operating characteristic curve.

^b^MCI: mild cognitive impairment.

^c^GBM: gradient-boosting machine.

^d^XGBoost: extreme gradient boosting.

## Discussion

### Principal Findings

This study used the KLoSA dataset and indicated that ML algorithms, particularly XGBoost, significantly outperformed conventional regression methods in predicting the onset of dementia, but the performance for predicting MCI was more modest. Like previous studies indicating that ML methods achieve relatively high accuracy (>0.80) compared with traditional methods [27,29,30], our results revealed that many models, including logistic regression and XGBoost, could achieve predictive performance levels similar to or exceeding those of previous studies.

Previous studies that incorporated traditional models for predicting the progression of MCI to dementia demonstrated accuracy levels comparable to our models. For instance, while earlier research reported Cox proportional hazards regression and gradient boosting models achieving similar accuracy levels, our findings indicated that for the MCI onset model, the logistic regression model outperformed all models, closely followed by XGBoost. For dementia onset prediction, the logistic regression model also exhibited superior performance, closely followed by XGBoost and Light GBM. These results suggest that ML models can achieve predictive performance levels similar to or exceeding those of previous studies. Large-scale systematic reviews comparing logistic regression and ML models for binary outcomes also observed that ML models have no performance benefit over logistic regression models in the clinical prediction of MCI or dementia, although ML is believed to perform better than traditional models when handling a larger number of potential predictors. Nevertheless, ML models are increasingly used in studies targeting older populations. In a recent systematic review, it was observed that approximately 40% of all published predictive analysis studies on dementia now incorporate ML algorithms, even though methodological limitations (lack of internal validation, such as bootstrapping) pervade in more than half of these studies [11]. ML models may be unnecessarily complex and more suitable for models with unconventional predictor variables such as imaging data [27]; however, it is possible that increasing causal insights will allow artificial intelligence to help with personalized interventions for dementia prevention in the near future. This potential is particularly relevant if “semisupervised methods,” incorporating both traditional statistical approaches and ML methodologies, use self-training in dementia risk prediction to address challenges associated with limited data entries in uncertain samples [29].

Our findings corroborate those of previous studies and highlight the protective role of higher sociodemographic factors against cognitive decline. Notably, our traditional model revealed that MCI onset was associated with lower educational attainment and economic well-being. In previous studies of South Korean older adults, similar findings reported that early-stage MCI patients with higher educational attainment and socioeconomic status have slower rates of cognitive decline in mental state examinations such as the Mini-Mental State Examination [30]. Contextually, it is important to consider that because South Korea experienced rapid economic development since 1970, educational attainment levels among older adults are significantly lower in Korean samples than in Western populations [31], with approximately 10.6% of older adults aged 65 years or older uneducated or illiterate, 31.7% with an educational attainment level of elementary school or less, and 23.3% with a level of middle school or less in 2020 [32]. From a sociohistorical perspective required for a full understanding of this age cohort [33], this may mean that the role of educational attainment in predicting cognitive decline is more prominent, as well as indicative of socioeconomic well-being or experience of economic hardship than other population groups [34]. Likewise, it is important to note that while 40 years of economic growth has resulted in decreased mortality rates by 70% to 80%, educational inequalities have increased or been stagnant, especially among Korean older populations [35], which may affect how these predictors play a role in health-related outcomes like MCI in this study.

In our ML model, we also identified associations between MCI onset and experiences such as everyday pain or being widowed. Additionally, the traditional model demonstrated significant relationships among the female sex, advanced age, and alcohol consumption with dementia onset. These findings underscore the importance of incorporating sociodemographic characteristics into early detection and prevention strategies for MCI and dementia.

This study reinforces the importance of higher educational attainment in cognitive health, particularly in individuals with early-stage MCI [30]. This suggests that education provides a cognitive shield through lifelong engagement, which is fundamental for building and maintaining cognitive reserves [36]. Specifically, education and engagement in mental activities such as solving jigsaw puzzles, playing cards, and reading books or newspapers have been shown to enhance memory and cognitive functions, thereby reinforcing the brain’s resilience against cognitive impairment as individuals age [37]. Additionally, this study highlights the significant impact of perceived economic well-being on MCI onset. Our findings align with a previous study showing that older adults who perceive their income as insufficient are nearly 30% more likely to develop cognitive impairment than their economically better-off counterparts [38]. This advocates higher socioeconomic status not just as a gateway to resources but also as an enabler of engagement in intellectually and socially enriching activities [39]. Moreover, we explored the influence of living alone as a potential factor limiting cognitive stimulation. Social connectivity is crucial for mental health and protects against cognitive decline, which underscores the negative effects of loneliness and social isolation [40]. Given that widowed individuals may face greater challenges in maintaining continuous communicative interactions than those living with others, fostering social connections emerges as a modifiable factor in mitigating the risk to cognitive health. However, in South Korea, approximately 20% of older adults are believed to live alone, while 58.4% live with a spouse, and 20.1% live with their children [32]. Relative to Western populations, like the United States, where approximately 28% of older adults live alone, including 21% of older men and 34% of older women [41], this difference may show that the negative impact of social contact on emotional well-being could be pronounced in the South Korea cohort due to cultural differences in social structure and family dynamics [42].

Our findings also align with those of a previous study indicating that advanced age and female sex significantly contribute to the risk of developing dementia, necessitating tailored interventions [43]. Furthermore, we delved into health behaviors and cognitive health, particularly highlighting the role of social engagement, including societal views on alcohol consumption. While previous studies have linked alcohol consumption to a reduced risk of cognitive decline [44,45], our findings suggest that this association may be more attributable to the social interaction inherent in drinking scenarios than to alcohol consumption itself [46]. Moreover, factors contributing to loneliness, such as living alone, social isolation, and widowhood, have been correlated with an increased risk of AD and dementia in older adults, especially when social networks lack meaningful connectedness and interaction quality [47]. However, delineating the precise relationship between alcohol consumption and dementia risk requires further research, considering various confounders such as genetics, daily lifestyle, and overall health [44,45].

Therefore, our findings highlight the paramount role of sociodemographic factors in the early prediction and prevention of cognitive impairments. This underscores the necessity of integrating sociodemographic features into community health care and public health strategies to address and mitigate cognitive decline more effectively. Additionally, this study emphasizes the need for an expanded implementation and study of ML algorithms that incorporate sociodemographic characteristics. By leveraging readily available sociodemographic data, we can provide targeted interventions for at-risk individuals, thereby enhancing cognitive resilience and reducing the incidence of cognitive impairments in an aging population. Further research on ML models that include sociodemographic features is essential to increase their applicability and effectiveness across diverse groups.

### Strengths and Limitations

This study has several notable strengths. To our knowledge, this is the first study to incorporate the KLoSA dataset to compare the predictive accuracy of ML and traditional statistical models for MCI and dementia onset. Furthermore, while many ML studies have methodological limitations regarding the lack of internal or external validation when predicting MCI [11], this study incorporated class weights to diagnose and address challenges associated with data imbalances and bias toward majority classes. We also attempted to discriminate between different levels of cognitive impairment by subdividing our population into MCI and dementia populations. Previous studies have reported that the lack of discrimination of dementia type is a methodological limitation in models that use ML methods for dementia risk prediction [11]. However, this study has some notable limitations. First, because our MCI (n=23) and dementia (n=70) samples were small, we were unable to perform traditional analyses on multiple variables such as drinking or smoking status, handgrip strength, comorbidity, and experience of falls, all of which may be important confounding variables affecting MCI or dementia onset among older adults. Likewise, one of the primary limitations of our investigation was the significant class imbalance in the dataset for the target variables “MCI” and “DEMENTIA.” The “MCI” variable had a ratio of approximately 215:1 (4952 non-MCI to 23 MCI), and the “DEMENTIA” variable had a ratio of approximately 70:1 (4905 nondementia to 70 dementia). Despite using class weighting to address this imbalance, our models were unable to correctly identify any true positive instances of MCI or dementia. This resulted in zero precision, recall, and *F*_1_-scores for the minority classes across all models, indicating that none of the models could accurately predict these conditions. The confusion matrices further reflected this limitation, showing that all true instances of MCI and dementia were classified as negatives (false negatives), with no instances classified as positives (true positives). The extreme class imbalance posed a significant challenge, limiting the effectiveness of our class balancing techniques and ultimately impacting the reliability of our predictive models for the minority classes. This limitation highlights the need for more balanced datasets in future studies or alternative strategies for dealing with severe class imbalances to improve model performance in predicting critical conditions such as MCI and dementia in the KLoSA dataset that was used in this investigation.

Second, although boosting mechanisms were used in our investigation, owing to their flexibility and capacity to reduce variance in smaller datasets, scholars have noted that gradient boosting techniques can often be overly sensitive, resulting in overfitting [48].

Third, due to the introduction of MCI in the KLoSA survey in 2018, this study was restricted to data from 2018 to 2020, which may limit the generalizability of our findings and the number of events observed, especially as most cognitive prediction papers use longer windows, that average 5+ years [49].

Also, the hyperparameters for each model were primarily set to default values, with specific parameters manually adjusted based on common practices within the literature. Systematic hyperparameter tuning methods, such as grid search or Optuna, were not used in this study.

Finally, the KLoSA dataset did not contain information on genetic biomarkers or environmental factors. However, an increasing number of studies have shown that genes such as *ADAMTS9*, *APOE*, *BDNF*, *CASS4*, *COMT*, *CR1*, *DNMT3A*, *REST*, and *TOMM40* are significantly correlated with cognitive aging and have the potential to enhance the predictive capability of ML models for cognitive decline [50]. The predictive performance would likely increase significantly in future studies that use Korean datasets if genetic and environmental biomarker data were incorporated into ML analyses. Genomic methods have been found to be effective in boosting predictive power in investigations of cognitive impairment [51] and MCI-to-AD progression [52].

### Conclusions

In this comparative study, which assessed various ML algorithms against traditional statistical methods for predicting MCI and dementia onset among older Korean adults, we observed that ML algorithms, particularly XGBoost, demonstrated promising potential in predicting MCI onset but not dementia onset. However, it is crucial to interpret these findings cautiously, given the relatively limited dataset and the absence of genetic biomarkers or certain environmental variables. Nevertheless, our methodology presents a viable auxiliary approach for identifying cohorts at high risk for MCI and dementia in community settings. Future investigations should acknowledge and leverage the predictive capacity of ML algorithms, such as by using boosting techniques, to forecast the onset of MCI and dementia among older adults. Additionally, further research efforts should aim to uncover and incorporate overlooked or unaccounted-for risk factors into predictive models.

## Appendix

Multimedia Appendix 1 TRIPOD+AI (Transparent Reporting of a Multivariable Prediction Model for Individual Prognosis or Diagnosis+Artificial Intelligence) checklist, list of features used in predictive models, and one-hot encoding information.

## Abbreviations

AD: Alzheimer disease
ADL: activities of daily living
AUC: area under the receiver operating characteristic curve
GBM: gradient boosting machine
IADL: instrumental activities of daily living
KLoSA: Korean Longitudinal Study of Aging
LTC: long-term care
MCI: mild cognitive impairment
ML: machine learning
SMI: subjective memory impairment
TRIPOD+AI: Transparent Reporting of a Multivariable Prediction Model for Individual Prognosis or Diagnosis+Artificial Intelligence
XGBoost: extreme gradient boosting

## Data Availability

The datasets analyzed during this study are available in the Korea Employment Information Service repository.
