# Supplementary material for: A Multivariable Prediction Model for Mild Cognitive Impairment and Dementia: Algorithm Development and Validation
Source: JMIR Med Inform. 2024 Nov 22;12:e59396. doi: 10.2196/59396 (PMC11624448; doi:10.2196/59396)
Supplement: Multimedia Appendix 1 [file medinform_v12i1e59396_app1.docx]

**Table S1.** TRIPOD+AI Checklist.

| **Section/Topic Item Development Checklist item/ evaluation^1^** | | | | **Reported on page** |
| --- | --- | --- | --- | --- |
| **TITLE** | | | |  |
| Title | 1 | D;E | Identify the study as developing or evaluating the performance of a multivariable prediction model, the target population, and the outcome to be predicted | Title |
| **ABSTRACT** | | | | |
| Abstract | 2 | D;E | See TRIPOD+AI for Abstracts checklist | Abstract |
| **INTRODUCTION** | | | | |
| Background | 3a | D;E | Explain the healthcare context (including whether diagnostic or prognostic) and rationale for developing or evaluating the prediction model, including references to existing models | **Page 5:** "Nevertheless, the increasing use of predictive models with machine learning (ML) algorithms that can process complex data...has encouraged researchers to investigate the potential of ML in identifying individuals with MCI and dementia..." |
|  | 3b | D;E | Describe the target population and the intended purpose of the prediction model in the context of the care pathway, including its intended users (e.g., healthcare professionals, patients, public) | **Page 5**: "The Republic of Korea presents a unique context for this study, with one of the fastest aging populations and the lowest birth rates globally..." |
|  | 3c | D;E | Describe any known health inequalities between sociodemographic groups | **Page 4:** “Adding to these challenges is the ethical dilemma for physicians in disclosing a dementia diagnosis, particularly among sociodemographic groups where factors such as cultural beliefs or socioeconomic status…” |
| Objectives | 4 | D;E | Specify the study objectives, including whether the study describes the development or validation of a prediction model (or both) | **Page 6:** "This study utilized KLoSA data, focusing on lifestyle factors, sociodemographic variables, and common tests, to compare the predictive accuracy of ML-based approaches with traditional models such as logistic regression." |
| **METHODS** | | | | |
| Data | 5a | D;E | Describe the sources of data separately for the development and evaluation datasets (e.g., randomised trial, cohort, routine care or registry data), the rationale for using these data, and representativeness of the data | **Page 4**: "This study utilized KLoSA data from 2018–2020. The KLoSA, established in 2000, is a biennial panel survey..." |
|  | 5b | D;E | Specify the dates of the collected participant data, including start and end of participant accrual; and, if applicable, end of follow-up | **Page 4**: "This study utilized KLoSA data from 2018–2020." |
| Participants | 6a | D;E | Specify key elements of the study setting (e.g., primary care, secondary care, general population) including the number and location of centres | **Page 4**: "The KLoSA, established in 2000, is a biennial panel survey..." |
|  | 6b | D;E | Describe the eligibility criteria for study participants | **Page 4:** "Participants aged 60 or older were included in the analyses for this investigation..." |
|  | 6c | D;E | Give details of any treatments received, and how they were handled during model development or evaluation, if relevant | **Page 6:** “For the computational analysis, 70% of the cases were randomly chosen for training…” |
| Data preparation | 7 | D;E | Describe any data pre-processing and quality checking, including whether this was similar across relevant sociodemographic groups | N/A |
| Outcome | 8a | D;E | Clearly define the outcome that is being predicted and the time horizon, including how and when assessed, the rationale for choosing this outcome, and whether the method of outcome assessment is consistent across sociodemographic groups | **Page 19**: “"MCI and dementia were identified based on the responses to the question…” |
|  | 8b | D;E | If outcome assessment requires subjective interpretation, describe the qualifications and demographic characteristics of the outcome assessors | N/A |
|  | 8c | D;E | Report any actions to blind assessment of the outcome to be predicted | N/A |
| Predictors | 9a | D | Describe the choice of initial predictors (e.g., literature, previous models, all available predictors) and any pre-selection of predictors before model building | **Page 21:** “The features included in our predictive models were selected based on their relevance to dementia and MCI prediction, informed by existing literature and expert…” |
|  | 9b | D;E | Clearly define all predictors, including how and when they were measured (and any actions to blind assessment of predictors for the outcome and other predictors) | **Page 20**: "Multiple sociodemographic variables including sex, age, educational attainment, living arrangements, marital status, and…” |
|  | 9c | D;E | If predictor measurement requires subjective interpretation, describe the qualifications and demographic characteristics of the predictor assessors | N/A |
| Sample size | 10 | D;E | Explain how the study size was arrived at (separately for development and evaluation), and justify that the study size was sufficient to answer the research question. Include details of any sample size calculation | **Page 21**: "For the computational analysis, 70% of the cases were randomly chosen for training…” |
| Missing data | 11 | D;E | Describe how missing data were handled. Provide reasons for omitting any data | **Page 19**: "Individuals without missing information on sociodemographic variables, including sex, homeownership, or self-reported diagnoses of MCI…” |
| Analytical methods | 12a | D | Describe how the data were used (e.g., for development and evaluation of model performance) in the analysis, including whether the data were partitioned, considering any sample size requirements | **Page 3**: "For the computational analysis 70% of the cases were randomly chosen for training whereas the remaining 30% were used as…” |
|  | 12b | D | Depending on the type of model, describe how predictors were handled in the analyses (functional form, rescaling, transformation, or any standardisation). | **Page 2**: "To prepare these features for the model, categorical variables were encoded using one-hot encoding. This approach…” |
|  | 12c | D | Specify the type of model, rationale^2^, all model-building steps, including any hyperparameter tuning, and method for internal validation | **Page 2**: "Multiple models including logistic regression, light gradient-boosting machine (GBM), XGBoost, CatBoost, Random Forest, Gradient Boosting, AdaBoost, Support Vector Classifier (SVM), and K-Nearest Neighbors (KNN)…” |
|  | 12d | D;E | Describe if and how any heterogeneity in estimates of model parameter values and model performance was handled and quantified across clusters (e.g., hospitals, countries). See TRIPOD_+_Cluster for additional considerations^3^ | **Page 3**: "To address longitudinal and sampling biases stemming from systemic sampling, the KLoSA provided weights for each wave ensuring…” |
|  | 12e | D;E | Specify all measures and plots used (and their rationale) to evaluate model performance (e.g., discrimination, calibration, clinical utility) and, if relevant, to compare multiple models | **Page 3**: "Metrics such as the area under the curve (AUC) and confusion matrix were calculated for each model to facilitate…” |
|  | 12f | E | Describe any model updating (e.g., recalibration) arising from the model evaluation, either overall or for particular sociodemographic groups or settings | **Page 2**: "Descriptive statistics were used to summarize the baseline sample…” |
|  | 12g | E | For model evaluation, describe how the model predictions were calculated (e.g., formula, code, object, application programming interface) | **Page 4**: "Python (version 3.7.2; Python Software Foundation) and the Scikit-learn library (version 0.20.2; David Cournapeau and Matthie…” |
| Class imbalance | 13 | D;E | If class imbalance methods were used, state why and how this was done, and any subsequent methods to recalibrate the model or the model predictions | **Page 9: “Class Imbalance Methods…”** |
| Fairness | 14 | D;E | Describe any approaches that were used to address model fairness and their rationale | **Page 3**: "These survey weights were incorporated into a PROC SURVEYLOGISTIC regression model, a procedure released in the SAS…” |
| Model output | 15 | D | Specify the output of the prediction model (e.g., probabilities, classification). Provide details and rationale for any classification and how the thresholds were identified | **Page 3**: "Metrics such as the area under the curve (AUC) and confusion matrix were calculated for each model to facilitate…” |

^1^ D=items relevant only to the development of a prediction model; E=items relating solely to the evaluation of a prediction model; D;E=items applicable to both the development and evaluation of a prediction model

^2^ Separately for all model building approaches.

^3^ TRIPOD+Cluster is a checklist of reporting recommendations for studies developing or validating models that explicitly account for clustering or explore heterogeneity in model performance (eg, at different hospitals or centres). Debray et al, BMJ 2023; 380: e071018 [DOI: 10.1136/bmj-2022-071018]

**Table S2.** List of Features Used in Predictive Models and One-Hot Encoding Information.

| **Feature Category** | **Feature Name** | **Description** | **One-Hot Encoded Columns** |
| --- | --- | --- | --- |
| **Demographic** | Age | Age in years | Age (Continuous) |
|  | Sex | Sex | Sex_Male, Sex_Female |
| **Socio-Economic** | Marital Status | Marital status | MaritalStatus_Married, MaritalStatus_Widowed, MaritalStatus_Single |
|  | Educational Attainment | Highest educational level attained | Education_Elementary, Education_Middle, Education_HighSchool, Education_University |
|  | Living Arrangements | Living situation | Living_Alone, Living_WithPartner, Living_With2OrMore |
|  | Region | Region of residence | Region_Rural, Region_Urban |
|  | Assets | Total assets | Assets (Continuous) |
|  | LTC Insurance | Awareness and use of long-term care insurance | LTCInsurance_Unaware, LTCInsurance_AwareNotInUse, LTCInsurance_InUse |
|  | Long-Term Care Home Service | Use of long-term care home services | LTCHomeService_Use, LTCHomeService_DoNotUse |
|  | Social Engagement | Frequency of social engagement | SocialEngagement_DailyWeekly, SocialEngagement_Monthly, SocialEngagement_YearlyOrLess |
| **Health** | ADL | Activities of Daily Living scores | ADL (Continuous) |
|  | IADL | Instrumental Activities of Daily Living scores | IADL (Continuous) |
|  | Handgrip Strength | Change in handgrip strength | Handgrip_IncreasedOrSame, Handgrip_Decreased |
|  | Drinking Status | Current drinking status | Drinking_Yes, Drinking_No |
|  | Smoking Status | Current smoking status | Smoking_Yes, Smoking_No |
|  | Frequent Exercise | Frequency of exercise | Exercise_Yes, Exercise_No |
|  | Obesity | BMI-based obesity status | Obesity_ObeseOverweight, Obesity_Normal, Obesity_Underweight |
|  | Comorbidity | Number of comorbidities | Comorbidity_None, Comorbidity_OneOrMore |
|  | Experience of Fall | History of falls | Fall_Yes, Fall_No |
|  | Pain in Everyday Life | Experience of pain | Pain_Yes, Pain_N |
